# Chromosome-level genome assembly of *Hippophae gyantsensis*

**DOI:** 10.1038/s41597-024-02909-w

**Published:** 2024-01-25

**Authors:** Mingyue Chen, Danni Yang, Shihai Yang, Xingyu Yang, Zhiyu Chen, Tianyu Yang, Yunqiang Yang, Yongping Yang

**Affiliations:** 1 School of Ecology and Environmental Science, Yunnan University, Kunming, China; 2 Plant Germplasm and Genomics Center, Kunming Institute of Botany, Chinese Academy of Sciences, Kunming, 650201 China; 3 Institute of Tibetan Plateau Research at Kunming, Kunming Institute of Botany, Chinese Academy of Sciences, Kunming, 650201 China; 4 Tibet Yunwang Industrial Corporation, Ltd., Shigatse, China; 5 University of Chinese Academy of Sciences, Beijing, 100049 China

**Keywords:** Plant sciences, Genome informatics

## Abstract

*Hippophae gyantsensis*, which is a native tree species in China, is ideal for windbreak and sand-fixing forests. It is an economically and ecologically valuable tree species distributed exclusively in the Qinghai-Tibet Plateau in China. In our study, we assembled a chromosome-level genome of *H. gyantsensis* using Illumina sequencing, Nanopore sequencing and chromosome structure capture technique. The genome was 716.32 Mb in size with scaffold N50 length of 64.84 Mb. A total of 716.25 Mb genome data was anchored and orientated onto 12 chromosomes with a mounting rate of up to 99.99%. Additionally, the genome was found to comprise approximately 56.84% repeat sequences, of which long terminal repeats(LTRs) that accounted for 33.19% of the entire genome. Meanwhile, a total of 32,316 protein-coding genes were predicted, and 91.07% of these genes were functionally annotated. We also completed a series of comparative genomic analyses to provide researchers with useful reference material for future studies on seabuckthorn.

## Background & Summary

*Hippophae gyantsensis*, which is a small tree of the family Elaeagnaceae, is an endemic species in the Qinghai-Tibet Plateau in China^1^. It is mainly distributed in the western part of the Qinghai-Tibet Plateau at an altitude range of 3,500–5,000 m^2^. Seabuckthorn plants have good drought resistance, barren resistance, saline-alkali resistance and cold resistance^3,4^. In addition, seabuckthorn has fast growth rate, strong reproduction and nitrogen fixation ability^5^. It is an excellent native tree species for windbreak, sand fixation and afforestation in Tibet and other places^6^. All seabuckthorn are rich in active substances, such as seabuckthorn flavonoids, vitamins, etc., they have great nutritional value^7–9^. Moreover, due to its unique geographical distribution and likely hybrid origin, *H. gyantsensis* is also an excellent species for studying the systematic geography of the Qinghai-Tibet Plateau^10^.

So far, the research on *H. gyantsensis* that have been conducted to date have mainly focused on its drought resistance, morphological characteristics, ecological distribution, and origin. Although the *H. gyantsensis* chloroplast genome has been published^11^ and phylogenetic trees have been constructed using partial nuclear sequences and chloroplast sequences^12–16^, because of a lack of whole-genome sequences, the genetic and evolutionary relationships of *H. gyantsensis* are still unclear^17–21^. For example, there is some controversy regarding whether *H. gyantsensis* is a hybrid of *Hippophae rhamnoides* Linn. subsp. *yunnanensis* Rousi and *Hippophae neurocarpa* or an independent species^2^. So a chromosome-level *H. gyantsensis* genome sequence will be a useful resource for research on the inheritance of *H. gyantsensis* and the genetic relationships of seabuckthorn. In this study, we assembled the *H. gyantsensis* whole-genome sequence and compared it with the genomes of 13 other representative plants.

## Methods

### Sample collection and genomic DNA sequencing

The *H. gyantsensis* plant used for the *de novo* genome assembly was collected in Jiangzi, Xizang province, China. High-molecular weight genomic DNA was extracted from the leaves according to the cetyltrimethylammonium bromide (CTAB) method^22^. Libraries were constructed by MGIEasy Universal DNA Library Prep Kit V1.0 (CAT#1000005250, MGI) following the standard protocol. The qualified libraries were sequenced on DNBSEQ-T7RS platform in GrandOmics Biosciences Co., Ltd. (Wuhan, China). Fastp v0.23.2^23^ was used to filter the raw Illumina sequencing data. ONT regular DNA were extracted using the Grandomics Genomic DNA Kit following the manufacturer’s guidelines. The quality of the extracted DNA was assessed and then long DNA fragments were recovered from the high-quality samples using the Blue Pippin system (Sage Science, Beverly, MA) by gel cutting. Then the Pippin HT system (Sage Science, USA) was employed to extract the size-selected long DNA fragments. After completing the damage repair and end repair steps, the 3’ ends of the recovered long DNA fragments were modified via the addition of A. The DNA was purified using magnetic beads prior to the ligation of a sequencing adapter using an SQK-LSK110 kit. Finally, the constructed DNA library was accurately quantified using a Qubit ® 3.0 fluorometer and added to the sequencing buffer. The solution was thoroughly mixed and then added to the Flow cell, which was transferred to the primed Nanopore PromethION sequencer for sequencing. A total of 55.86 Gb clean Illumina short-read data and 102.9 Gb Nanopore read data were generated.

### Hi-C sequencing

For chromosome conformation capture (Hi-C) sequencing, genomic DNA was extracted for the Hi-C library from H. gyantsensis, we constructed the Hi-C library and obtained sequencing data via the DNBSEQ-T7RS platform. Firstly, Cells were treated with 2% final concentration fresh formaldehyde to induce the formation of crosslinks (DNA–protein and protein–protein), after which the cells were lysed and samples were extracted and assessed in terms of quality. The high-quality samples were used for the ‘Hi-C fragment’ preparation process. Specifically, a restriction endonuclease was used to digest chromatin. The effect of this digestion was determined. After the biotin labeling, blunt end ligation, and DNA purification steps, the Hi-C samples were prepared. The DNA quality was evaluated and the high-quality DNA was retained. A standard library construction procedure was completed, which was followed by the optimization of PCR conditions and amplification. The amplified products were sampled for the ‘Hi-C fragment connection point quality control test’ before the construction of the sequencing library was completed. The library that passed the quality control step was sequenced using the DNBSEQ-T7RS platform. Finally, 183 Gb Hi-C data was generated.

### Transcriptome sequencing

After extracting the total RNA from samples in CTAB-LiCI method^22^, the eukaryotic mRNA was enriched from total RNA using Dynabeads mRNA Purification Kit (Cat#61006, Invitrogen). The mRNA was fragmented using fragmentation reagent in MGIEasy RNA Library Prep Kit V3.1 (Cat# 1000005276, MGI). The first cDNA strand was synthesized using random hexanucleotide primers and mRNA as the template, after which the second cDNA strand was synthesized by adding buffer, dNTPs, RNase H, and DNA polymerase I. Following an end repair step, a poly(A) tail and a sequencing adapter were added. The cDNA fragments were amplified by PCR and purified with MGIEasy DNA Clean beads (CAT#1000005279, MGI). The MGIEasy Circularization module (CAT # 1000005260, MGI) was used to perform thermal denaturation and circularization of the double stranded PCR products. The single stranded circle DNA (ssCirDNA) was formed as the final library for sequencing on the DNBSEQ-T7RS platform. A total of 34.23 Gb data was generated.

### Chromosome-level genome assembly

**Fig. 1:**
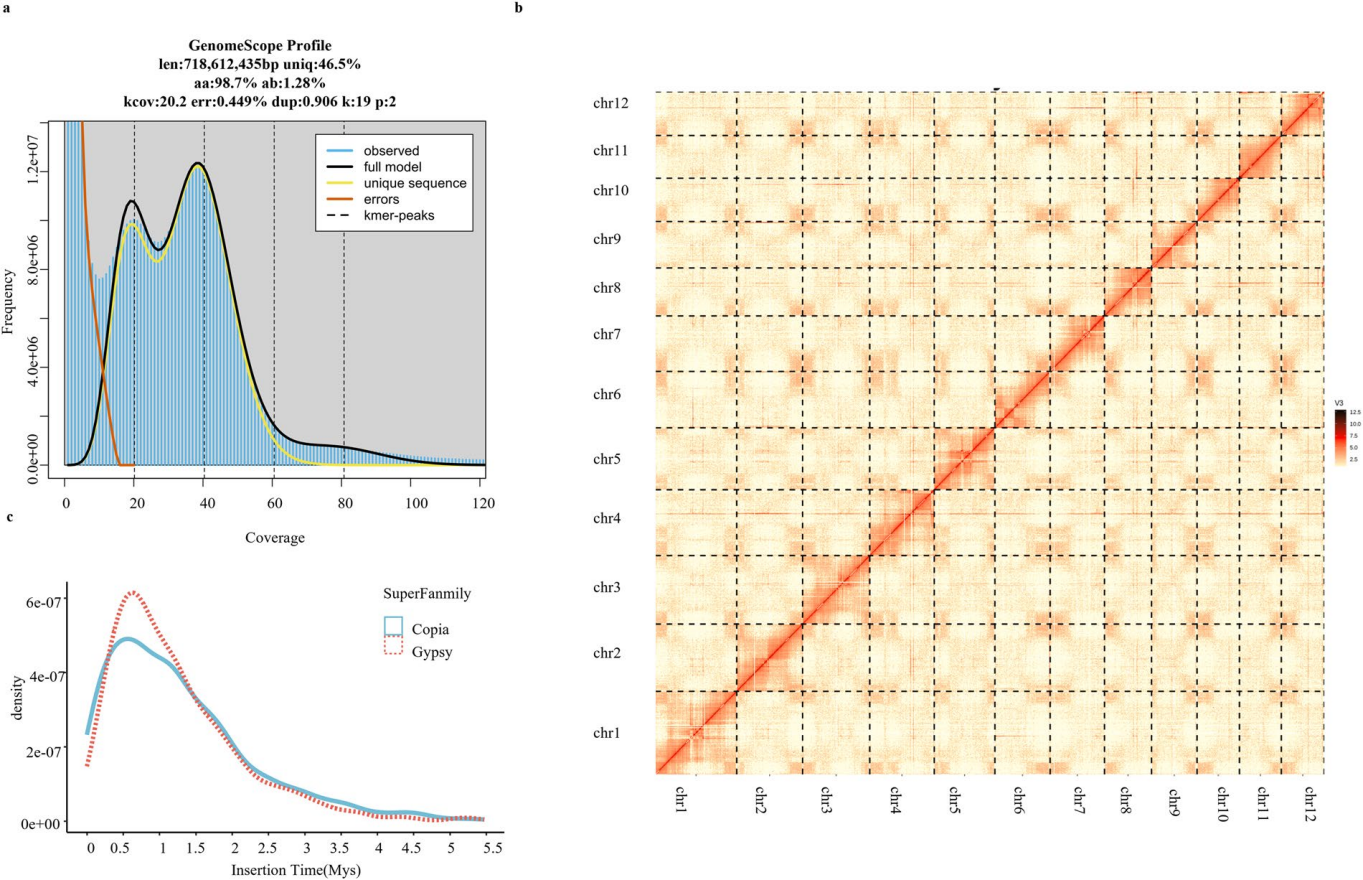
Chromosome-level genome assembly of the *H. gyantsensis* (**a**) K-mer analysis of *H. gyantsensis*. (**b**) Hi-C interaction heatmap for the *H. gyantsensis* genome. (**c**) Distribution of LTR -RTs insertion times for *H. gyantsensis*.

We performed a k-mer analysis of the *H. gyantsensis* genome to select the appropriate genome assembly procedure. Jellyfish v2.3.0^24^ was used to analyze the clean Illumina data and determine the k-mer frequency distribution. Finally, GENOMESCOPE v2.0^25^ was used to estimate the genome size and heterozygosity according to the frequency distribution structure. The estimated genome size was 718.61 Mb, with a heterozygosity of 1.28% (Fig. 1a).

We combined the clean Illumina and Nanopore reads for the *de novo* genome assembly. Specifically, NextDenovo v2.4.0 (https://github.com/Nextomics/NextDenovo) was used to initially assemble the Nanopore data and then NextPolish v1.4.1^26^ and the filtered Illumina data were used to correct the assembled structure. PurgeDup v1.2.5^27^ was used to eliminate redundancy and generate the final haploid assembly. Then we used the ragtag^28^ script to rearrange the chromosomes of the previous assembly results with the *H. rhamnoides* genome as a reference. Finally, Juicer was used to align the Hi-C reads to the assembled draft genome and then the default parameters of 3D-DNA^29^ were used to map the contigs to the chromosome-level scaffolds (Fig. 1b). Chromosome identification numbers and orientations were refined according to the previously published *H. rhamnoides* genome^30^ and named chr1 to chr12. We assembled a chromosome-level *H. gyantsensis* genome with a total scaffold length of 716 Mb. Additionally, its 12 chromosomes accounted for 99.99% of the total length. The contig N50 of the genome assembly was 23 Mb and the scaffold N50 was 64 Mb. We evaluated the genome quality using Benchmarking Universal Single-Copy Orthologs (BUSCO v5.4.5)^31^. The results indicated the genome was 98.8% complete. Furthermore, 1,614 expected embryophyta genes were identified (Table 1).

**Table 1:** Details regarding the genome assembly and annotation.

Genome assembly	
Genome size(Mb)	716
Assembly level	Chromosome
Chromosome number	12
Contig N50(bp)	23152489
Saffold N50(bp)	64845084
GC content(%)	29.72
Number of genes	32316

### Repeat and gene annotation

Repeats were annotated using Extensive *de novo* TE Annotator (EDTA v2.1.2)^32^, which is a comprehensive tool that integrates multiple prediction tools. After obtaining the TE library annotated by EDTA, TEsorter v1.3^33^ was used to reclassify ‘LTR-unknown’ and then deepTE was used to classify test.tesorter.unk. Finally, we combined the three obtained TE databases and determined that 56.84% of the *H. gyantsensis* genome consisted of repetitive elements, which were primarily long terminal repeats (LTRs) (33.19%) and terminal inverted repeats (TIRs) (15.71%) (Table 2). We also analyzed the timing of the LTR insertions in the *H. gyantsensis* genome. A total of 2,481 full-length Long terminal repeat-retrotransposons (LTR -RTs) were inserted over a period of approximately 1 million years (Fig. 1c).

**Table 2:** Annotation of repeat elements in the *H. gyantsensis*.

Type	Precent(%)
DNA_transposon	1.38%
LTR	33.19%
TIR	15.71%
Low_complexity	0.10%
NonLTR	2.58%
Helitron	2.70%
Repeat_region	1.18%
Total	56.84%

### Gene annotation

**Fig. 2:**
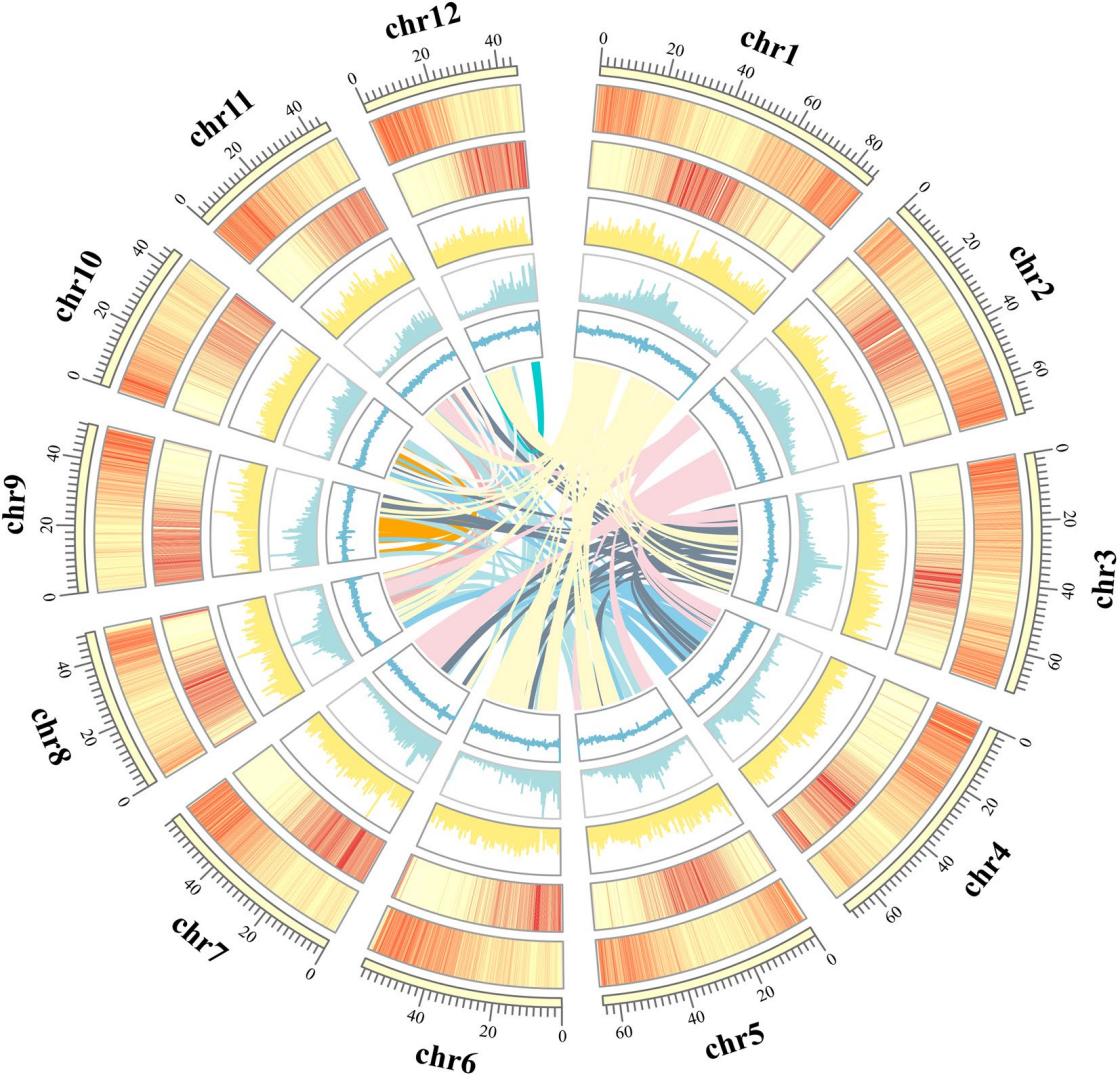
Circos plot of the genomic landscape of *H. gyantsensis*. (**a**) Gene density. (**b**) repeat sequences density. (**c**) Gypsy density. (**d**) Copia density. (**e**) GC content. (**f**) Interspecies collinearity.

To annotate genes, we first used RepeatMasker v4.1.2-pl^34^ and the TE library to mask the whole-genome sequence. Gene annotations were performed by integrating evidence from homology-, De novo- and transcriptome-based information. Braker v3.03^35^ were employed for the gene structure annotation.Then the annotation results as the input of Maker2^36^ for an additional annotation to obtain a higher quality model. A total of 32,316 genes were predicted, with an average gene length of 3,541 bp. TBtools^37^ was used to visualize the gene density, GC content, Gypsy density, Copia density, and chromosomal synteny of 12 chromosomes (Fig. 2).

Genes were functionally annotated in two ways. First, DIAMOND v2.0.15^38^ was used to align protein sequences with the sequences in the NCBI non-redundant protein (NR) and Swiss-Prot databases^39^ Second, eggNOG-mapper^40^ was used to annotate protein sequences according to the Kyoto Encyclopedia of Genes and Genomes (KEGG)^41^ Gene Ontology (GO)^42^ and Pfam databases. Thus, 91.07% of the predicted genes were annotated on the basis of at least one of these databases (Table 3).

**Table 3:** Functional annotation of *H. gyantsensis* genes.

Genome annotation	Number of elements
predicted protein-coding genes	32316
Swissprot	22776
GO	15941
KEGG	13942
Pfam	25674
NR	29430
Tatol	29431

### Gene family evolution analysis

**Fig. 3:**
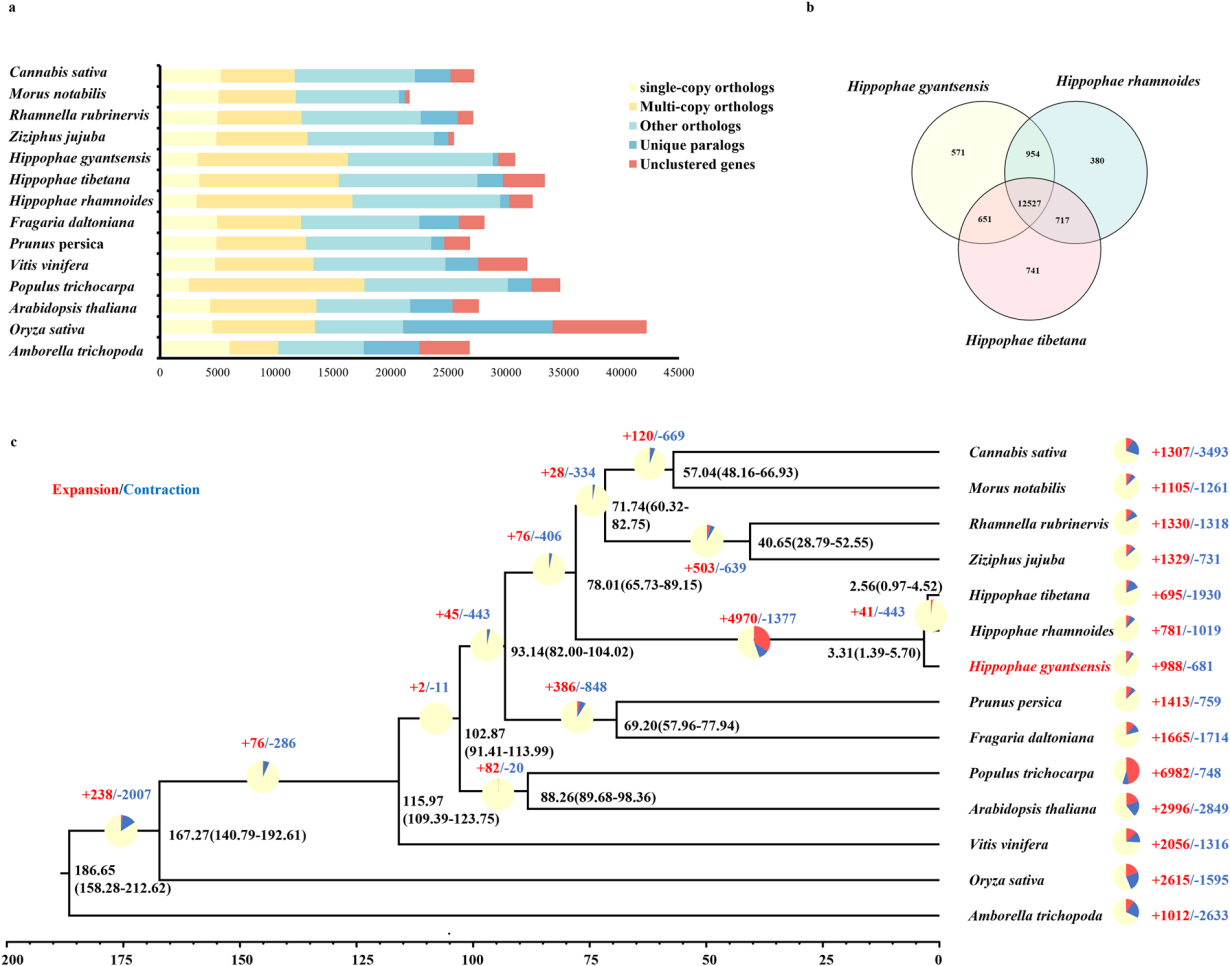
Gene family evolution analysis (**a**). Clusters of gene families from *H. gyantsensis* and other species. (**b**) Venn diagram of OGs shared by the three seabuckthorn species. (**c**) Divergence time tree of *Hippophae gyantsensis* and 13 other species. The number of expanded gene families (red) and the number of contracted gene families (blue) are indicated to the right of each species branch The yellow/red/blue circles and the corresponding numbers reflect the expansion (remain or gain) or contraction (loss) of gene families in specific species.

Gene families were analyzed using protein sequences from *H. gyantsensis* and 13 other plant species (*H. rhamnoides*, *Amborella trichopoda*, *Arabidopsis thaliana*, *Cannabis sativa*, *Hippophae tibetana*, *Morus notabilis*, *Oryza sativa*, *Populus trichocarpa*, *Fragaria daltoniana*, *Rhamnella rubrinervis*, *Vitis vinifera*, *Ziziphus jujuba*, and *Prunus persica*). The default parameters of OrthoFinder v2.5.4^43^ were used to identify orthogroups in the 14 species. A total of 416,260 genes from the 14 species were classified into 27,387 gene families (Fig. 3a). There were no significant differences in the number of gene families among the three *Hippophae* species. However, these three species had more gene families than the other analyzed species, with the exception of *V. vinifera*. The results of the analysis of the gene families in the three seabuckthorn species were visualized using a Venn diagram^44^ Fig. 3b). These three species shared 12,527 homologous gene families, but 517 gene families were unique to *H. gyantsensis*.

The 596 single-copy genes that were identified in the 14 species using OrthoFinder were used to construct phylogenetic trees. The single-copy homologous gene coding sequences were extracted using the seqkit tool^45^ and aligned with MUSCLE v3.8.31^46^ Next, the seqkit tool was used to connect the sequences to form supergenes, after which trimAL v1.4.rev15^47^-gt 0.6 -cons 60) was used to trim the supergene sequences. The trimmed sequences were used to construct the phylogenetic tree according to the maximum likelihood method using RAxML^48^

The MCMCtree program in the PAML v4.10.6 package^49^ as used to estimate the divergence time for each node in the phylogenetic tree. Divergence times in the TimeTree database^50^(http://www.timetree.org) were used as calibration time-points, including the divergence times for *V. vinifera* and *P. persica* [109.8–122.4 million years ago (mya)], *F. daltoniana* and *P. persica* (49.1–77.1 mya), and *C. sativa* and *M. notabilis* (48.9–70.9 mya). The phylogenetic tree with divergence times and the sorted gene family results were used as the input to construct the phylogenetic tree with gene family expansion and contraction information using the CAFÉ V5 program^51^ According to the constructed phylogenetic tree (Fig. 3c), H. *gyantsensis* (family: Elaeagnaceae) diverged from *Z. jujuba* (family: Rhamnaceae) and *M. notabilis* (family: Moraceae) approximately 78.01 mya. Notably, the phylogenetic tree indicated that *H. gyantsensis* diverged first among the three *Hippophae* species. Moreover, *H. tibetana* and *H. rhamnoides* are more closely related to each other than to *H. gyantsensis*. Extensive gene family expansions and contractions occurred after the divergence of the genus *Hippophae*.

### Identification of WGD events and analysis of genome collinearity

**Fig. 4:**
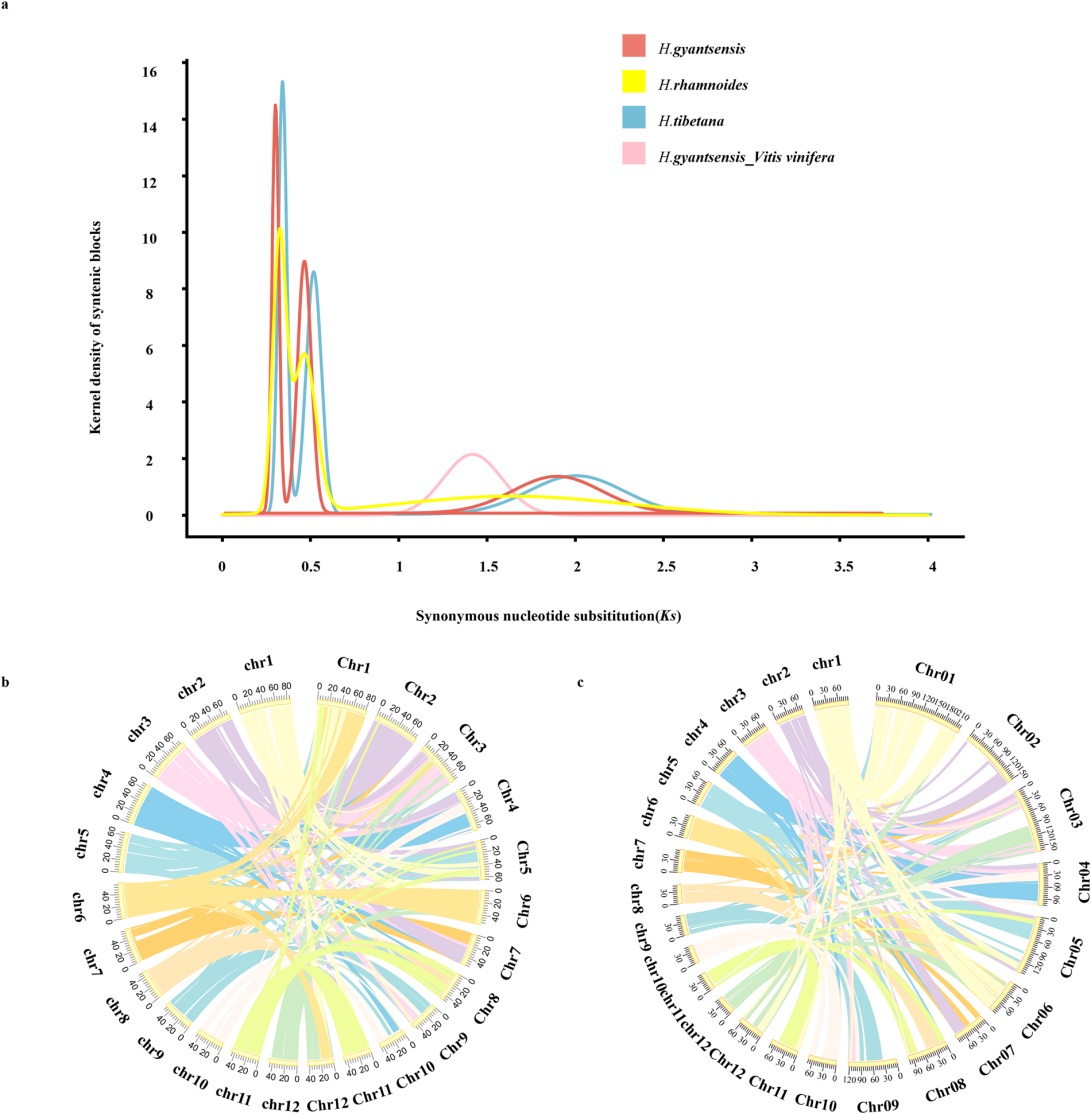
WGD event and collinearity analysis of *H. gyantsensis*. (**a**) Distribution of the number of synonymous substitutions per synonymous site (Ks) confirming the occurrence of a whole-genome duplication (WGD) event in *H. gyantsensis*. (**b**) Whole-genome synteny between *H. gyantsensis* and *H. rhamnoides* (**c**) Whole-genome synteny between *H. gyantsensis* and *H. tibetana*.

We used the WGDI toolkit (v0.6.4)^52^ to detect WGD events. DIAMOND v2.0.15^38^ was used to identify homologous genes (e-values no higher than 1e-5). The WGDI toolkit was also used to identify collinear genes (parameter: ‘-icl’). The ‘ks’ parameter in WGDI was modified to calculate the KS values, whereas the ‘bi’ and ‘c’ parameters were modified to screen the collinearity results (ks-col = ks_YN00). Finally the ‘kp’ parameter was modified to calculate the KS peak. The results were visualized using the ‘kf’ parameter (Fig. 4a). Three collinear block peaks (0.315 ± 0.001, 0.483 ± 0.002, and 2.029 ± 0.002) were detected for *H. gyantsensis*. Three collinear block peaks were also detected for *H. rhamnoides* and *H. tibetana*, which was consistent with the results of previous studies that suggested two lineage-specific polyploidization events occurred in the genus *Hippophae* within a relatively narrow timeframe ^30,53^.

In this study, MCscan (Python version)^54^ was used for the genome-wide collinearity analysis involving *H. gyantsensis*, *H. tibetana*, and *H. rhamnoides*. In addition, JCVI^55^ and TBtools^37^ were used to draw collinearity figures according to the gene collinearity between species. Chromosome 2 of *H. tibetana* differed significantly from the corresponding chromosomes in *H. gyantsensis* and *H. rhamnoides* (Fig. 4b,c).

## Data Records

The genomic Illumina sequencing data were deposited in the Sequence Read Archive at NCBI SRR25382505^54^.

The genomic Nanopore sequencing data were deposited in the Sequence Read Archive at NCBI SRR25382499^56^ and SRR25382498^57^.

The transcriptome Illumina sequencing data were deposited in the Sequence Read Archive at NCBI SRR25382500-SRR25382503^58–61^. The Hi-C sequencing data were deposited in the Sequence Read Archive at NCBI SRR25382504^62^. The final chromosome assembly were deposited in the GenBank at NCBI JAUQSU000000000^63^.

The final gene structure annotation, repeat annotation, and gene functional prediction were deposited in the Figshare database^64^.

## Technical Validation

### DNA quantifcation and qualification

For all sequencing samples, whether DNA samples or RNA samples, we performed sample quality testing, the detailed steps have been mentioned in the Method section

### Assessment of genome assemblies

In addition to using Benchmarking Universal Single-Copy to evaluate genome quality(mentioned in method section), we used BWA-MEM2 v2.2.1^65^ to align Illumina short reads to the *H. gyantsensis* genome to evaluate the accuracy of the final genome assembly. Analysis showed that 97.84% of the short reads were successfully mapped to the *H. gyantsensis* genome. We further assessed the base quality of genome assembly by estimating the quality value score (QVS) using Inspector version 1.0.1^66^, which showed a high QVS of 33.18. These fndings indicate that the quality of our assembled genome is high. In addition, the LAI package inside the LTR_retriever v.2.9.0^67^ is used to evaluate the LTR Assembly Index, and the result file of EDTA is used as input. The results showed that the LAI value of the genome was 11.7. In summary, the genome can provide a good reference for subsequent work.

## Data Availability

nextDenovo: input_type = raw, read_type = ont, read_cutoff = 1k, seed_cutoff = 34747, sort_options = -m 20 g -t 14, minimap2_options_raw = -t 14, pa_correction = 8, correction_options = -p 14, minimap2_options_cns = -t 14, minimap2_options_map = -t 14, nextgraph_options = -a 1 NextPolish: sgs_options = -max_depth 100 -bwa, lgs_options = -min_read_len 1k -max_depth 100, lgs_minimap2_options = -x map-ont TEsorter: -db rexdb-plant Repeatmasker: -pa 14 -s -xsmall Blastp: E-value ≤ 1e-5 Swiss-Prot: E-value ≤ 1e−5 Nr: E-value ≤ 1e−5 Orthofinder: -S diamond -M msa -T fasttree trimAl: -gt 0.6 -cons 60 RAxML: raxmlHPC-PTHREADS -m PROTGAMMAJTT -f a -p 123 -x 123 -# 100 Wgdi: pvalue = 0.05 Other commands and pipelines used in data processing were executed using their corresponding default parameters.
